# Editorial

**Published:** 1917-03

**Authors:** Edmund Vance Cooke


### EDITORIAL

MRS. F. E. DANIEL, Publisher and Managing Editor.

Did you tackle the trouble that came your way
With a resolute heart and cheerful?
Or hide your face from the light of day
With a craven soul and fearful?
O, a trouble's a ton, or a trouble's an ounce,
Or a trouble is what you make it,
And it isn't the fact that you're hurt that counts,
But only, how did you take it?

You are beaten to earth. Well, well, what's that?
Come up with a smiling face.
It's nothing against you to fall down flat,
But to lie there, that's disgrace.
The harder you're thrown, why the higher you bounce;
Be proud of your blackened eye!
It isn't the fact that you're licked that counts,
It's how did you fight—and why?

And though you be done to the death, what then?
If you battled the best you could,
If you played your part in the world of men,
Why, the Critic will call it good.
Death comes with a crawl, or comes with a pounce,
And whether he's slow or spry,
It isn't the fact that your'e dead that counts,
But only, how did you die?

*—Edmund Vance Cooke.*

